# Material research from the viewpoint of functional motifs

**DOI:** 10.1093/nsr/nwac017

**Published:** 2022-02-12

**Authors:** Xiao-Ming Jiang, Shuiquan Deng, Myung-Hwan Whangbo, Guo-Cong Guo

**Affiliations:** State Key Laboratory of Structural Chemistry, Fujian Institute of Research on the Structure of Matter, Chinese Academy of Sciences, Fuzhou 350002, China; State Key Laboratory of Structural Chemistry, Fujian Institute of Research on the Structure of Matter, Chinese Academy of Sciences, Fuzhou 350002, China; State Key Laboratory of Structural Chemistry, Fujian Institute of Research on the Structure of Matter, Chinese Academy of Sciences, Fuzhou 350002, China; Department of Chemistry, North Carolina State University, Raleigh, NC 27695-8204, USA; State Key Laboratory of Structural Chemistry, Fujian Institute of Research on the Structure of Matter, Chinese Academy of Sciences, Fuzhou 350002, China

**Keywords:** functional motif, functional motif arrangements, microscopic structures, structure–property relationship

## Abstract

As early as 2001, the need for the ‘functional motif theory’ was pointed out, to assist the rational design of functional materials. The properties of materials are determined by their functional motifs and how they are arranged in the materials. Uncovering functional motifs and their arrangements is crucial in understanding the properties of materials and rationally designing new materials of desired properties. The functional motifs of materials are the critical microstructural units (e.g. constituent components and building blocks) that play a decisive role in generating certain material functions, and can not be replaced with other structural units without the loss, or significant suppression, of relevant functions. The role of functional motifs and their arrangement in materials, with representative examples, is presented. The microscopic structures of these examples can be classified into six types on a length scale smaller than ∼10 nm with maximum subatomic resolution, i.e. crystal, magnetic, aperiodic, defect, local and electronic structures. Functional motif analysis can be employed in the function-oriented design of materials, as elucidated by taking infrared non-linear optical materials as an example. Machine learning is more efficient in predicting material properties and screening materials with high efficiency than high-throughput experimentation and high-throughput calculations. In order to extract functional motifs and find their quantitative relationships, the development of sufficiently reliable databases for material structures and properties is imperative.

## Teaser

Material microscopic structures were analyzed from the viewpoint of ‘functional motifs’, which could be employed to assist the rational design of functional materials, build quantitative structure-property relationships, and accelerate material research period.

## INTRODUCTION

Materials provide the basis for manufacturing and play a fundamental role in technical revolution. In searching for new materials, the trial-and-error method has been used traditionally. Given the relatively low efficiency of this method, the time between the discovery of a new material and its practical application is typically 30 years or more. This time-consuming and costly process hardly meets the urgent need to discover advanced materials. The entire process of material research generally involves several stages, which include initial material discovery, accumulation of experimental and theoretical data, determination of structure–property relationships, and prediction and optimization of material performances. Several effective methods and powerful tools, such as high-throughput experimentation and high-throughput calculations, must be employed to accelerate this process. Given that the properties of materials are determined by their structures, the key to realizing a rational design of materials with desired performance is to understand the detailed information about their structures (e.g. constituent components, building blocks, geometrical arrangements and symmetry). In 2001, the authors proposed the development of the ‘functional motif theory’ in an attempt to assist the rational design of functional materials, build rules of quantitative structure-property relationship and achieve the final goal of shortening the material research period [1]. The term ‘functional motifs’ refers to the critical microstructure units (e.g. constituent components and building blocks) that play a decisive role in generating certain material functions. These units can not be replaced with other structure units without the loss, or significant suppression, of relevant functions. The properties of materials are determined by their functional motifs and how these are arranged in the materials, with the latter determining the quantitative structure–property relationship.

In the first 10 years of the 21st century, several large databases and websites on material structures and properties were built and developed rapidly. Afterwards, US President Barack Obama announced the Materials Genome Initiative (MGI) in 2011, a project with a mission to discover, develop, manufacture and deploy advanced materials twice as fast, at a fraction of the current cost, by the interplay of high-throughput experiments, computation and digital data sharing [2]. One and a half years later, the Chinese version of the MGI was launched, creating a quantitative component–structure–performance relationship that is crucial for transforming material design and production from the traditional trial-and-error method to a scientific method [3]. Several related scientific research projects were launched in China; these studies include major research plans for ‘function-guiding structure design and controllable synthesis of crystalline materials’ (2016) and ‘basic research of high-performance functional materials with the structural ordering of functional motifs’ (2019) by the National Natural Science Foundation of China, and the Strategic Priority Research Program of the ‘structure and function guiding the creation of new substances’ by the Chinese Academy of Sciences in 2016.

The hierarchy of material structure involves information crossing multiple lengths and time scales. In terms of the length scale of structural features, material structure can be classified into macroscopic, mesoscopic and microscopic structures. Macroscopic structures in material science refer to those with sizes >∼1 μm in length. The gradient structure is a type of special structure that exhibits a systematic change in microstructure along the depth on a macroscopic scale. It can be widely found in many biological systems, such as bones and plant stems, in which structures change gradually from the surface to the interior, and have the advantage of maximizing physical and mechanical performance while minimizing material cost [4]. The properties of macroscopic structures are well described by classical mechanics. Microscopic structures refer to structural features on a length scale of <∼10 nm, which is equivalent to approximately dozens of repeat unit cells in inorganic crystalline materials. The fundamental structural units resolvable in microscopic structures include lattices, chemical bonds, atoms, spins and charge density. Microscopic structures strongly affect intrinsic material properties such as superconductivity, ferroelectricity, magnetism and non-linear optical (NLO) properties [5–7], which require quantum mechanics to describe. The length scale of mesoscopic structures (ranging from ∼10 nm to 1 μm) lies between those of macroscopic and microscopic structures. The important structures in this length scale include interfaces, domains and superlattices that can be created by artificial methods. The III–IV semiconductor superlattices exhibit interesting transport and electrical properties associated with quantum size effects, while multi-wavelength second harmonic generation (SHG) and coupled parametric processes can be realized with high efficiency in quasi-periodic dielectric superlattices [8].

The intrinsic properties and functions of materials are mostly determined by the microscopic structures, while the mesoscopic and macroscopic structures play an important role in generating new extrinsic functions that are not inherent to the materials but enhance their intrinsic properties. Microscopic structures and intrinsic properties are the bases for further development of new functions of advanced materials via artificial modulation of mesoscopic and macroscopic structures. In this article, we focus on microscopic structures and intrinsic properties. In principle, the structure–property relationships of functional materials can be elucidated on the basis of functional motifs and the quantitative relationships between them, which depend strongly on how they are arranged in the materials. These relationships allow eventual achievement of the function-oriented design of new functional materials. Over hundreds of years, many types of functional materials have been studied by employing experimental and theoretical methods, and massive data on material structures and properties have been accumulated. For a particular structural feature and property (or function), only one main structure–property relationship is expected in general. However, several different interpretations are possible for a given structure–property relationship. Thus far, no general guideline has been presented on how to extract functional motifs from materials, and their crucial arrangements, to establish the structure–property relationships from massive data on functional materials.

**Scheme 1.**
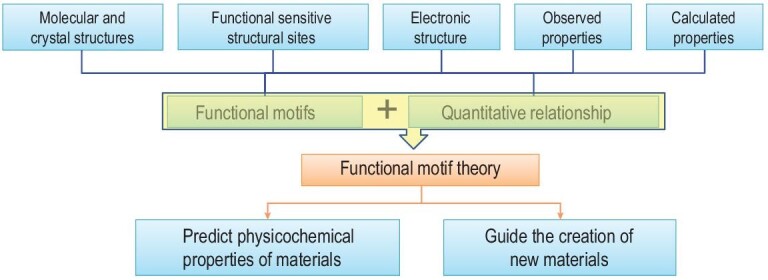
Functional-motif-based material research paradigm.

Given the importance of microscopic structures in material science, a new research paradigm was suggested for material studies from the point of view of functional motifs (Scheme 1). This paradigm starts with the main aspects of microscopic structures, including molecular structures of materials, crystal structures of crystalline materials, electronic structures governing all material properties and function-sensitive structural sites, which are structural units associated with material properties and functions. Molecular and crystal structures are mainly determined by experimental methods, while electronic structures are determined by theoretical calculations. The properties and functions of materials can be evaluated by experimental measurements and/or by theoretical calculations. On the basis of the microscopic structures and properties of materials, the functional motifs crucial for the material properties can be extracted, the quantitative relationships between them can be investigated and the results could be further developed as the ‘functional motif theory’. The latter should be useful as a guideline for creating new materials and as a tool for predicting the physicochemical properties of materials.

The following sections explore the functional motifs of materials as follows: first, microscopic structure is classified into six classes based on the short- and long-range ordering of different structural elements, and their main fundamental structural aspects and intrinsic material properties are described. Second, the structural features and related structure–property relationships in microscopic structures are discussed. For each type of microscopic structure, the main structural features and parameters, the methods with which to describe and characterize them, and the general structure–property relationships for several representative material examples are elucidated. Then, infrared (IR) NLO materials are considered as one way of introducing the function-oriented design strategy of new functional materials, in which the role of functional motifs of materials is stressed in the design of materials. This strategy differs from the traditional structure-oriented design strategy. Next, the important role of high-throughput experimentation and calculation in material studies and the challenges of extracting functional motifs from a huge amount of data on material structures and properties are discussed. Finally, remarks on the functional motifs of materials are summarized.

## CLASSIFICATION OF MATERIAL MICROSCOPIC STRUCTURES

The microscopic structures of materials on a length scale smaller than ∼10 nm with maximum subatomic resolution largely involve the arrangements of atoms, chemical bonds, spin moments, charge density distributions, distortions and atomic defects. Different types of microscopic structures are present in various materials, from glassy-state solids with no long-range order to three-dimensional (3D) crystalline materials with translational symmetry. The microscopic structures of materials may be discussed in terms of six types: (i) crystalline structures possessing a long-range order of atoms, (ii) magnetic structures with a long-range order of spin moments in crystalline materials, (iii) aperiodic structures with long-range organized atom modulations from crystalline materials, (iv) defect structures with long-range random or non-random distributions of atomic defects in crystalline materials, (v) local structures representing local-coordination environments of atoms in the range of several coordination shells and (vi) electronic structures representing electron density distributions in real space (or position space) and those representing electron distributions in momentum space (or *k*-space). It should be noted that although the features of aperiodic, defect and local structures may have the same physical origin, such as atomic displacement and replacement from the perfect crystal structure, they present different aspects of structure distortion and affect material properties in different ways. Such a classification scheme is beneficial to the extraction of functional motifs and to understanding the quantitative structure–property relationships of materials.

**Figure 1.**
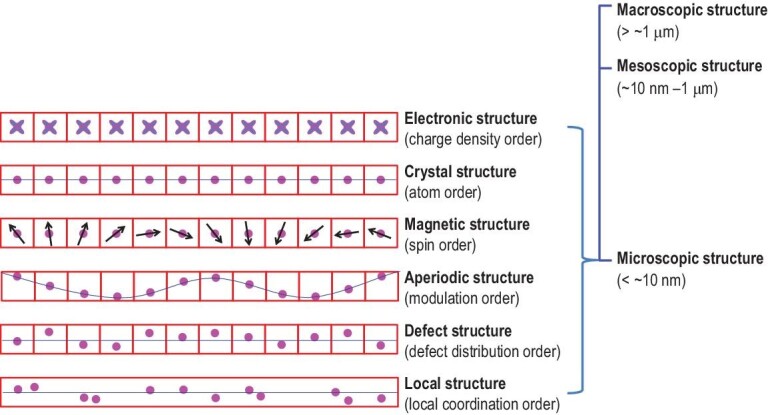
Structure hierarchy and classification of microscopic structures. Pink balls in red squares represent the atoms in a repeat unit cell; black arrows represent spin moments. The blue lines highlight the relative positions of atoms.

Figure 1 schematically depicts the differences and relationships between the basic structural units of these microscopic structures. X-ray, neutron and electron diffraction (or scattering) measurements are the typical methods for characterizing microscopic structures. In most cases of microscopic structures obtained experimentally, no information is obtained regarding any non-periodic structure of the sample used for measurements because they are averaged. In general, several different types of microscopic structures may exist simultaneously in the same material, but a limited number of them usually act as main contributors, determining the performance of the material. In general, each microscopic structure is closely related to certain material properties. The lattice and magnetic phase diagrams of a magnetic material are predominantly determined by its crystal and magnetic structures, respectively. Table 1 summarizes the typical structural features involved in each type of microscopic structure and their closely related properties. Frequently, different microscopic structures are related to each other, and they synergistically determine the properties of a material. The electric conductivities of semiconductors are determined by electronic and defect structures. Magnetic–electric couplings in multiferroics are determined by their crystal and magnetic structures.

**Table 1.** Representative structural information and associated material examples of microscopic structures.

Structure types	Primary information	Material examples
Electronic structure	Electron (charge) density distribution, energy levels (bands), wavefunction, density matrix, etc.	Electrical and optical properties, superconductivity, topological insulator, etc.
Crystal structure	Atom positions, occupancies and thermal vibrations, symmetry, etc.	Mechanical, optical, thermal, electrical and magnetic properties, non-linear optic, phase transition under external fields, ferroelectricity, anisotropic electrical conductivity, electrical permittivity, Young's modulus, etc.
Magnetic structure	Magnetic moment of ions, spin order, propagation vector, symmetry, etc.	Magnetism, multiferroicity, etc.
Aperiodic structure	Long-range modulated deviations of atom positions, occupancies and thermal vibrations, symmetry, modulation wave vector, etc.	Non-linear conductivity, etc.
Defect structure	Long-range distribution order of defects, vacancies, interstitials, stacking faults, etc.	Electric conductivity, catalysis, superionic conductivity, electrochemical performance, etc.
Local structure	Local (short-range) structure features of bond types, lengths and concentrations, etc., in amorphous, pharmaceutical, optoelectronic, electrically/magnetically ordered materials.	Chemical activity, negative thermal expansion, etc.

## STRUCTURAL FEATURES AND RELATED STRUCTURE–PROPERTY RELATIONSHIPS OF MATERIAL MICROSCOPIC STRUCTURES

### Crystal structure

The most common microscopic description of a crystalline material is the crystal structure, whose constituents (e.g. atoms, ions or molecules) are arranged in a highly ordered way, forming a crystal lattice that extends in all directions. A crystal structure is normally determined by least-square refinements of the atom parameters (i.e. the atom positions, their occupancies and the thermal vibration factors) against the X-ray, neutron or electron diffraction data of single-crystal or powder samples.

**Figure 2.**
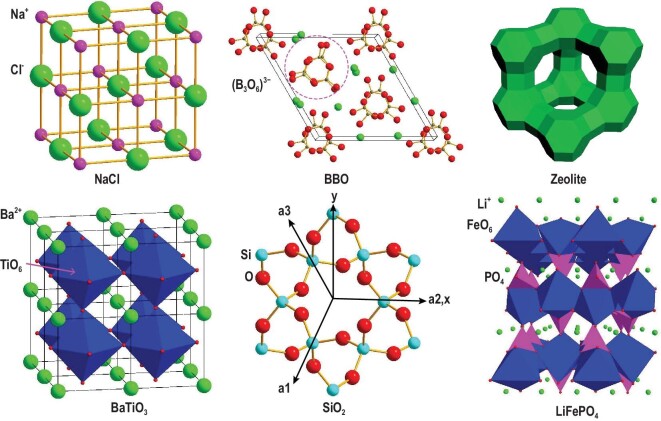
Crystal structures of NaCl, β-BaB_2_O_4_ (BBO), zeolite, BaTiO_3_, quartz SiO_2_ and LiFePO_4_ containing different types of functional motifs. NaCl is one of the most useful materials for general-purpose spectroscopic windows, and its optical transparency is mainly determined by the close noble-gas electron configurations of Na^+^ and Cl^–^ ions forming the face-centered cubic arrangement. BBO exhibits excellent ultraviolet-visible-near-IR NLO properties, which are mainly determined by (B_3_O_6_)^3−^ units with non-centrosymmetric arrangements. Zeolites are 3D microporous crystalline materials, whose separation properties are mainly determined by their constituent ions with arrangements that form continuous tunnels. BaTiO_3_ exhibits a strong room-temperature ferroelectricity, which originates from distorted TiO_6_ units. Quartz SiO_2_ is a well-known piezoelectric material, whose piezoelectricity is mainly determined by the electric dipole moments (Si-O pairs) with non-centrosymmetric arrangements. LiFePO_4_ is a typical cathode material for lithium-ion batteries, whose charge and discharge properties are mainly determined by the synergetic role of the porous structure and variable valence of iron ions.

In the crystal structures of materials, the basic building blocks (e.g. atoms, ions and molecules) repeat to generate the associated structural patterns (e.g. clusters, coordination polyhedra, rings, chains and layers) as a result of the formation of chemical bonds and the minimization of the overall energy of the condensed structure. The structure of a crystalline material can be identified as the functional motif for a certain property if it is indispensable for such property. The aromatic molecules (e.g. fluorene and pyrene) and metal ions (e.g. Mn^2+^ and rare earth ions) of several phosphor materials undergo electronic transition from the ground state to excited state under ultraviolet light or an electric field, providing dominant contributions to luminescent properties [9]. Thus, these aromatic molecules and metal ions form the functional motifs of the phosphor materials. Metal chalcogenide tetrahedra MQ_4_ (M = Zn, Cd, Ga, In, Ge; Q = S, Se) are typical building blocks of some IR NLO compounds, and they are the main source of NLO efficiency. Thus, MQ_4_ tetrahedra act as functional motifs of IR NLO materials [10]. In contrast to the case of phosphor and IR NLO materials, in which certain structural units play a role in constructing the crystal structures and generating material properties, voids, tunnels and interstices formed by structural units may also be crucial for material properties. Therefore, structural units forming voids, tunnels and interstices should be considered as functional motifs in a material. The tunnels in fast-ion conductors provide the ion migration pathways, and they are important for the realization of ionic conductivity and electrochemical properties [11]. Figure 2 shows several well-known materials with different structural units as their corresponding functional motifs.

Crystalline materials are characterized by translational symmetry, which leads to a long-range structural order, and by the point groups and space groups, which provide information about the symmetry of atomic arrangements in a repeat unit cell. The symmetry of atomic arrangement is crucial for certain physical properties. Almost all covalent and ionic bonds possess non-zero electric dipole moments. However, ferroelectric properties are found only in those compounds if the electric dipole moments of the bonds form a long-range order to have a non-zero overall moment in one direction and if the direction of this overall moment can be reversed when the direction of the external electric field is switched. Therefore, the arrangement of bond electric dipole moments plays a decisive role in ferroelectric properties. A material exhibits piezoelectricity, i.e. it accumulates electric charge under stress or strain, when it has a non-centrosymmetric arrangement of its electric dipole moments.

Crystalline materials could present a large number of physical and chemical properties when flexible structure units are combined with various arrangements of different symmetries. One of the main features of crystalline materials is anisotropy. This property is significantly different from that of glasses. Various crystal properties (e.g. electrical conductivity, dielectric constants and Young's modulus) may vary in different directions of a crystal. Several properties are closely related to the crystal’s structural features (Table 1). A crystal structure has a long-range order of structural units with a certain point-group symmetry. The breaking of a certain symmetry element or a change in the basic structural units results in other types of microscopic material structures.

### Magnetic structure

**Figure 3.**
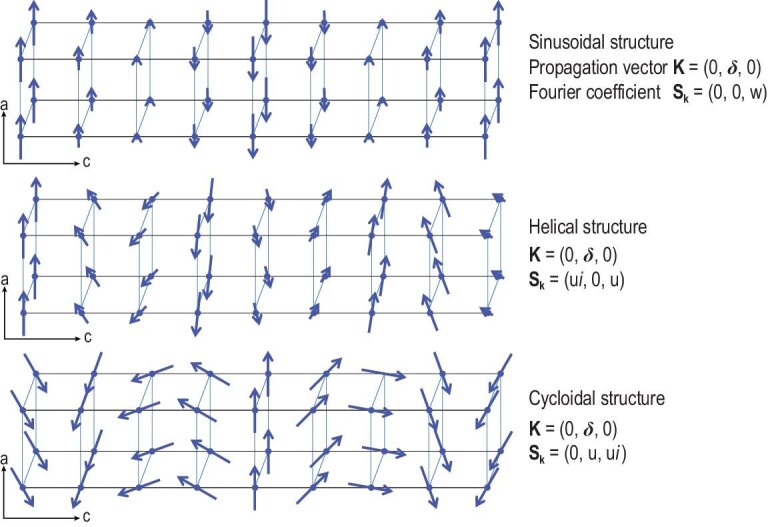
Schematic of a spin density wave, a helix and a cycloid that all occur in a magnetic material with moderate spin frustration [12]. The magnetic structures are simulated using anisotropic magnetic coupling configurations with the help of McPhase [94].

A magnetic structure can only be present in a structure with at least one atom with a permanent magnetic moment. Ordered magnetic structures refer to ordered arrangements of static magnetic moments (mostly spin moments) in a crystal lattice, which occur below a particular temperature (i.e. magnetic phase transition temperature). They can be described by magnetic space groups for commensurate structures, or more generally by commensurate or incommensurate propagation vectors. Magnetic orderings are relatively simple if they consist of identical magnetic ions, and if the coupling between adjacent spin moments is collinear, that is, either parallel (ferromagnetic) or antiparallel (antiferromagnetic). Magnetic structures become more complicated when the involved arrangements are non-collinear (e.g. helical or spiral, cycloid and conical spin orders) [12] and when two or more different moments are involved. Figure 3 shows typical sinusoidal, helical and cycloid structures. In a cycloid or a helix, the magnetic moment is identical in magnitude at all magnetic ions but its direction is different from one magnetic site to another along a certain chain direction. In a sinusoidal structure, known as a spin density wave (SDW), the magnetic moments are collinear but their magnitudes vary sinusoidally from one magnetic site to another along a certain chain direction. Such magnetic structures occur in magnetic materials with spin frustration, i.e. in which not all spin arrangements can be made collinear. When the extent of spin frustration is moderate, a magnetic system adopts a non-collinear spin arrangement, such as a cycloid or a helix, in which the successive spins along a chain rotate by a certain angle. The plane of the spin rotation is parallel to the chain direction in a cycloid, but perpendicular to the chain in a helix. Either a cycloid or a helix is chiral [13,14] because the spin rotation can be either clockwise or counterclockwise along the chain. The resulting two magnetic structures are opposite in chirality but identical in energy. When they occur with equal probability below a certain temperature *T*_SDW_, the superposition of the two chiral structures takes place, forming an SDW. When the temperature decreases to below *T*_SDW_, the electronic structure of a material may be relaxed to energetically favor one of two chiral structures, thereby leading to a cycloid or a helix. If a magnetic material is strongly spin frustrated, that is, the magnetic sites are subject to competing or contradictory constraints, it cannot adopt a long-range magnetic order but exhibits spin-liquid and spin-glass behavior because the ground magnetic state is represented by a large number of different spin configurations. The magnetic structure of a material with a long-range order can normally be determined by neutron diffraction or X-ray resonant magnetic scattering measurements on single crystal or powder samples.

Magnetic moments and their order are the main features of magnetic structures. The properties of magnetic materials (e.g. paramagnetism, ferromagnetism and antiferromagnetism) are closely related to magnetic structures. Ferromagnetic materials, such as transition metals (Fe, Co, Ni), rare earths with 64 ≤ Z ≤ 69 and alloys of ferromagnetic elements (MnBi and Cu_2_MnAl) can become permanently magnetized. The ferromagnetism of materials is mainly determined by spin moments and their aligned arrangements. Magnetoelectric compounds, such as CuO, CuBr_2_, BiMnO_3_, BiFeO_3_ and RMnO_3_ (R = Ho–Lu, Y), are types of multiferroics that exhibit magnetism and ferroelectricity simultaneously. They form when a special spin order (e.g. cycloidal and E-phase antiferromagnetic spin orders) breaks the inversion symmetry, thereby giving rise to ferroelectricity [15]. Therefore, spins of magnetic ions and electric dipole moments of cation–anion pairs are the functional motifs of magnetoelectric materials. The magnetic and ferroelectric orders and their interplay play a crucial role in governing magnetoelectric properties.

### Aperiodic structure

Translational symmetry is the key property characterizing crystalline materials and crystal structures. However, a long-range order in crystalline materials can be achieved by methods different from translational symmetry, resulting in aperiodic crystals, which include incommensurately modulated structures, incommensurate composites and quasicrystals [16,17]. Modulated and composite crystals have atomic structures that can be described as variations of periodic structures, while quasicrystals differ fundamentally from crystals possessing translational symmetry.

**Figure 4.**
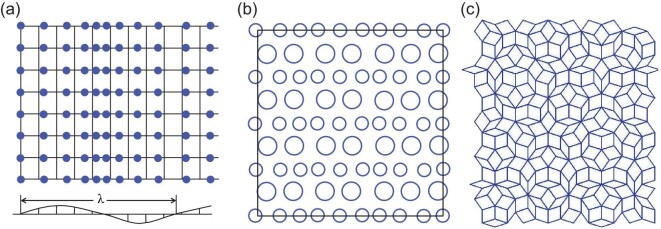
Schematic of aperiodic structures. (a) Crystal with incommensurate displacive modulation, in which atom positions deviate periodically from those of a conventional periodic crystal. (b) 2D model of an incommensurate composite. (c) 2D Penrose tiling quasicrystal constructed by a skinny rhombus with an angle of 36° and a fat rhombus with an angle of 72°. The schemes are based on those in ref. [17].

A modulated structure is characterized by a density (or atom arrangement) that obeys a space–group symmetry with a finite density variation (or finite displacement of each atom), which can be described by a modulation function and a wave vector. A modulated crystal is commensurate if the length of the modulation vector is a rational multiple of the basic length of periodicity in the direction of the modulation vector and incommensurate if it is an irrational multiple. A commensurate modulated structure can be described using a supercell of the crystal structure. If the modulation consists of deviations from the basic structure in the positions, then the modulation is displacive (Fig. 4a). When the probability distribution deviates from that of the basic structure, then the modulation is occupational. Modulated structures frequently exist in compounds that exhibit phase transitions under temperature variation, as in NaNO_2_, K_2_SeO_4_ and LiAlSiO_4_. Such phases occur as intermediates between a low-temperature phase of low symmetry and a high-temperature phase of high symmetry.

An incommensurate composite structure is based on two interpenetrating periodic lattice structures that are mutually incommensurate (Fig. 4b). This structure can be relatively easily formed in misfit layer compounds such as [LaS]_1.14_[NbS_2_] [18]. Other possibilities for constructing incommensurate composite crystals include arranging two collinear columns of atoms, each with their own periodicity, along the chain direction, as in [Sr]_1+x_[TiS_3_] (x ≈ 0.1) [19].

Quasicrystals have distinct property and diffraction patterns that manifest 5-fold, and other non-crystallographic point symmetries. Two types of quasicrystals are known. The first type is polygonal (dihedral) quasicrystals, which have a local 8-, 10- or 12-fold rotation symmetry (octagonal, decagonal or dodecagonal quasicrystals, respectively). They are periodic along the axes and quasiperiodic in planes normal to them. The second type is icosahedral quasicrystals, which are aperiodic in all directions [20]. A simple method of constructing two-dimensional (2D) quasicrystals is to employ a skinny rhombus with an angle of 36° and a fat rhombus with an angle of 72°. A 2D Penrose tiling with quasicrystal structure can be formed when specific special matching rules are fulfilled (Fig. 4c) [21]. Quasicrystals exist universally in many metallic alloys (Al-Li-Cu, Al-Mn-Si, Al-Ni-Co, Al-Pd-Mn, Al-Cu-Fe and Al-Cu-V) and polymers [22].

Aperiodic structures can be described mathematically as a cut through super space and their reciprocal space is a projection of the 3 + *n*-dimensional reciprocal super space onto 3D reciprocal space, where *n* represents the number of modulation vectors. Meanwhile, different types of modulation functions, such as harmonic wavefunctions, are needed to improve the description of aperiodic structures with various distortions. Although diffraction datasets of different dimensions are used, the process of determining an aperiodic structure is similar to that of determining a periodic crystal structure. In general, satellite diffractions resulting from a modulated structure are much weaker than the main diffractions resulting from a crystal structure. Thus, strong X-ray or electron sources are necessary for the diffraction measurements of aperiodic structures.

Aperiodic crystals have been observed in many crystalline materials, including minerals, metals, alloys (quasicrystals), organic and inorganic materials, and macromolecules. Several materials exhibit properties that depend strongly on aperiodic long-range order, which may be viewed as a functional motif in materials. Upon lowering the temperature, a certain metal reaches a charge density wave (CDW) state, in which the charge distribution exhibits a modulation to that of the metallic state. The CDW formation, which introduces an insulating energy gap at the Fermi level of the metallic state, is driven by the electron–phonon interactions associated with the nested Fermi-surface of the metal [23,24]. Metals can also reach a superconducting state. Superconductivity is similar to CDW, in that it also introduces an energy gap at the Fermi level as a result of the forming of Cooper pairs responsible for superconductivity. This gap prevents the superconducting state from reverting to the metallic state unless the temperature is raised. Superconductivity competes with CDW [25]. The competition has been found in several transition-metal chalcogenides (e.g. NbSe_2_ [26], HfTe_3_ [27], ZrTe_3−x_Se_x_ [28] and YBa_2_Cu_3_O_y_ [29]). Several NLO compounds, such as Cs_2_TB_4_O_9_ (T = Ge, Si) [30] and A_2_SnS_5_ (A = Ba, Sr) [31], have aperiodic structures, where aperiodic structural modulation may be beneficial to their SHG efficiency. In the case of A_2_SnS_5_ (A = Ba, Sr), a 44% or 25% distortion of the Sn/S building units can lead to a significant SHG enhancement compared with α-Ba_2_SnSe_5_ without modulation. Aperiodic structures can also exist in other material systems such as relaxor ferroelectric Ca_0.24_Ba_0.76_Nb_2_O_6_ and Ca_0.31_Ba_0.69_Nb_2_O_6_ [32] and multiferroic CaMn_7_O_12_ with low Cu-doping [33].

### Defect structure

Although perfect crystals are fully described by a set of translation and basis vectors, real-world materials are never perfect, because defects and thermal vibrations introduce non-negligible deviations from the ideal order. Crystallographic defects, which commonly exist in real crystals or crystalline materials, perturb the regular patterns of perfect crystals. Crystallographic defects can be classified into point, line, plane and bulk defects. Point defects include interstitial atoms, substitutional atoms and vacancies. Linear defects (dislocations) refer to areas where the atoms are out of position in the crystal structure. The two basic types of dislocations are edge and screw dislocations. Edge dislocation is caused by the termination of a plane of atoms in the middle of a crystal. In such a case, the adjacent planes bend around the edge of the terminating plane. Thus, the crystal structure is perfectly ordered on either side. Screw dislocation is a structure in which a helical path is traced around a linear defect (dislocation line) by the planes of atoms in the crystal lattice. Most dislocations are probably a hybrid of edge and screw dislocations. Planar defects are mainly produced by a disruption of the long-range stacking sequence. The three types of planar defects are stacking fault, twin region and grain boundaries. Grain boundaries occur where the crystallographic direction of the lattice abruptly changes. Bulk defects occur on a notably larger scale than other types of crystal defects. Voids and precipitates are typical bulk defects.

**Figure 5.**
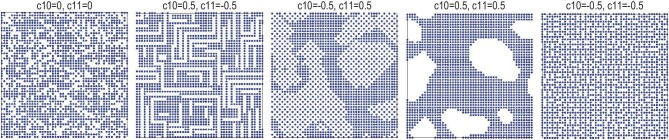
2D models using 50 × 50 cells (one atom in each cell) with different correlation coefficients (c10 and c11) between nearest neighboring atoms along the cell axis [(1, 0), (0, 1), (−1, 0) and (0, −1) directions for c10] and diagonal directions [(1, 1), (−1, 1), (1, −1) and (−1, −1) directions for c11]. The zero, positive and negative values of c10 and c11 are for random, positive and negative correlation cases, respectively. The structure models were simulated by using the DISCUS package [36].

Defect structures are more complicated and different from other material microscopic structures. Thus, the parameterization and determination of defect structures are difficult to unify and differ significantly from those of other structure types [34]. In principle, any parameters suitable for the description of defect structure models can be used for structural simulation and refinement. They include the commonly used correlation coefficients (or short-range parameters) and neighboring probabilities [35]. Structural defects are usually described by average structural parameters, such as split atom positions, mixed or partial occupancies and abnormal temperature factors. However, structures with the same average structural parameters may have different distributions of positions and occupancies. With partial occupancy as an example (Fig. 5), the crystallography-equivalent positions with identical vacancy of 30% may have different distribution patterns resulting from varied correlation coefficients between neighboring atoms along different directions. The structural features of different distribution patterns may occasionally lead to significantly varied material properties. Instead of the single algorithm of least-square fitting usually employed in the refinement of other microscopic structures, reverse Monte Carlo and differential evolutionary (or genetic) algorithms are frequently used in the determination of defect structures. Analyses of pair distribution function pattern and total (X-ray or neutron) scattering measurements of single crystals and powdery samples are normally used for defect structures, and they differ from the commonly used diffraction data for other structure types.

Numerous properties such as the lifetimes of charge carriers in semiconductors are determined by defects. Properties that may appear as the intrinsic properties of a material, including the specific resistance of semiconductors, the conductance in ionic crystals, or diffusion properties, are dominated by defects. In addition, defects strongly affect the behavior of phonons and electrons and they play an important role in the modulation of thermal and electric properties of thermoelectric materials [36]. Numerous defects (e.g. cracks, wrinkles and domain boundaries) extensively impair the electrical transport properties of graphene sheets prepared by chemical vapor deposition, leading to undesirable fluctuations in device performance [37]. Defects also play a key role in determining the electrochemical performance of electrode materials. LiTMO_2_ (TM = Ni, Mn, Co, or Ni_x_Mn_y_Co_z_ with *x* + *y* + *z* = 1) is an important cathode material for high-energy-density lithium-ion batteries. In the structure of the cathode, the Ni/Li disorder (or Ni/Li exchange) usually exists and has a detrimental effect on the Li diffusivity, cycling stability, first-cycle efficiency and overall electrode performance when the concentration is high. However, a low concentration of Ni/Li disorder can improve the thermal and cycling structure stability of Ni-rich LiTMO_2_ materials [38]. Therefore, in these defect-dominated functional materials, the defect structure should be their functional motif.

### Local structure

Studies on crystalline materials emphasized the periodic arrangement of atoms. This periodicity and its underlying symmetry have effects on the formal description of properties such as the spectra of electronic energies and vibrational frequencies. By contrast, studies on liquids and glasses can only focus on local structures, i.e. arrangements of atoms surrounding individual atoms in the range of several coordination shells. Local structures are important for a wide range of condensed matter, and they may govern the functions of certain materials, including negative thermal expansion materials, amorphous materials and shape memory alloys [39].

Given that local structures do not repeat periodically, they can not be determined by the standard

methods for atomic structure analysis, namely, normal X-ray diffraction, neutron diffraction and electron diffraction techniques. One important method for local structure determination is total scattering analysis [40]. Determining the structures of crystals and materials with local distortions involves measuring the intensities of X-ray or neutron beams scattered from the material. However, such studies differ with regard to the manner in which the data are analyzed. For crystalline materials, most of the scattering is in the form of intense Bragg peaks, and the arrangements of atoms within the repeating structure can be obtained via Fourier transform. For materials with local distortions, an analysis of the complete diffraction pattern is required because the analysis of the integrated Bragg reflection intensity yields information on the strictly averaged crystal structure only. This analysis can be performed in reciprocal space by an analysis of diffuse scattering, or after a Fourier transform into Patterson-space (either in 3D or in 1D projection, as in analyses of powder diffraction and pair distribution function patterns). In addition, muon spin resonance, X-ray absorption spectroscopy (extended X-ray absorption fine structure and X-ray absorption near-edge structure) and surface scanning microscopy are effective methods for detecting local structures.

A local structure usually coexists with other microscopic structure types with long-range order in crystalline materials, whose properties may be sensitive to the local structures. Most recent functional materials are impure, but they often possess doped elements for various functions. In certain circumstances, local structures dominantly contribute to the properties of materials. In the semiconductor industry, a small amount (∼0.1% or less) of dopant added to a pure Si crystal can result in a p- or n-type semiconductor.

**Figure 6.**
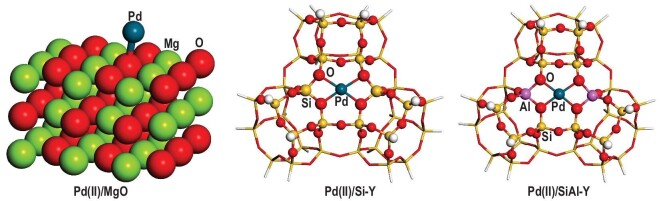
Optimized geometries of model-supported Pd(II) catalysts with local structure around Pd atoms. Reproduced from ref. [42] with permission. Copyright 2019, American Chemical Society.

In various solid catalysts used in the chemical industry, specific metal atoms serve as the most active sites of catalytic reactions. Thus, the atomic structures surrounding the active sites, that is, the localized impurity sites of catalysts, must be determined. Pd(II)-based catalysts are often used to selectively generate dimethyl carbonate (DMC). First-principle calculations indicate that the mononuclear-isolated local structure of Pd(II) centers is critical for the selective formation of DMC [41]. Figure 6 shows the local structure of mononuclear-isolated Pd(II) centers anchored on the supports of Y-type silica-alumina and silica zeolites and MgO. The three local structures have different electron affinities and energy gaps between the HOMO and LUMO, resulting in different stabilities of Pd(II)-supported catalysts [42]. The local structures surrounding the dopant and active-site atoms are important for semiconducting and catalytic properties and they can be called functional motifs of the corresponding catalysts.

### Electronic structure

The electronic structure of a crystalline material is generally represented by the electron density distribution in real space or by the electronic band structure in *k*-space. Details of the electronic structure are typically obtained by first-principles calculations. However, it can also be measured experimentally by using quantum crystallography techniques such as multipolar, constrained wavefunction and density matrix refinements [43–45]. The methods in quantum crystallography have been widely used in various systems, from inorganic and molecular materials to proteins, leading to the possibility of gaining insight into the physical and chemical properties of a material from its tiny crystal [46,47]. The electronic band structure can be obtained by angle-resolved photoemission spectroscopy measurements.

The electron density distribution of a material is usually characterized by the electron density *ρ*, the gradient of the electron density $\nabla {\rm{\rho }}$, the Laplacian of electron density ${\nabla ^2}\rho $ and the associated atom and bond topological properties based on the quantum theory of atoms in molecules [48]. Material properties and functions are related to certain features of the electron density distribution. The Laplacian of *ρ* means a local accumulation of electron density if ${\nabla ^2}\rho < 0$ (as in a Lewis base or nucleophile) and a local depletion of electron density if ${\nabla ^2}\rho > 0$ (as in a Lewis acid or electrophile). Therefore, the Laplacian distribution can be used to locate possible sites of nucleophilic attack and predict the bonding characteristics of chemical reactivity.

**Figure 7.**
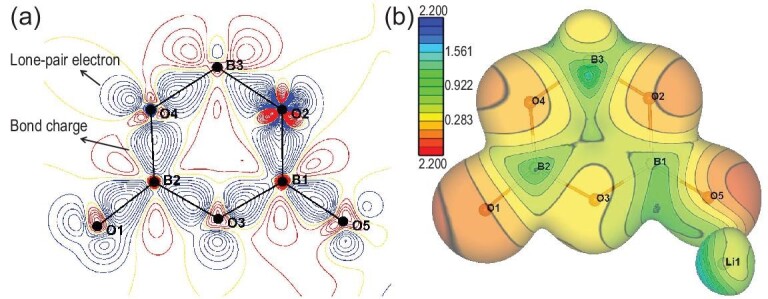
(a) Static deformation electron density and (b) electrostatic potential maps of the asymmetric unit of LBO. Data were obtained by multipole refinement of X-ray diffraction data using Mo Kα radiation (λ = 0.71073 Å) at 85 K.

The ionic and covalent characters of chemical bonds can be evaluated from the static deformation electron density, which is the difference between the multipole-refinement density and the superposition of independent atom densities, without considering the thermal smearing effect. Independent atom density is the electron density of an isolated atom with no chemical bonding. The deformation electron density highlights the areas in which the electron density deviates strongly from the superposed atom densities, namely, the lone-pair regions and the bonds between atoms. LiB_3_O_5_ (LBO) is one of the most widely used NLO crystals in ultraviolet/visible/near-IR regions. The lone-pair electrons of the O atoms and the bond charges on the B–O covalent bonds can be observed from the static deformation electron density map (Fig. 7a) of the asymmetric unit of LBO. However, no evident charge density can be found between Li and O atoms. These observations indicated that B-O and Li-O interactions are mainly covalent and ionic, respectively. The corresponding electrostatic potential map (Fig. 7b) shows that the boron and oxygen atoms have positive and negative net charges, respectively, consistent with the classic B–O bonding character. The valence electron of the lithium atom is negligible and it has a net positive charge. The non-linear optical functional motif of LBO was uncovered by *in-situ* electron density and wavefunction studies under laser irradiation [49].

Electronic structure calculations are carried out in real space for discrete molecules and in *k*-space for condensed matter with periodicity. They provide information about electron density distribution, the occupied and unoccupied energy states, and the spin moments and their preferred orientations. All properties of materials essentially originate from their electronic structures and the complex interactions between the electrons and nuclei. The electronic structure determines the cohesive energy, which in turn determines the structure. The transport, optical, magnetic and superconducting properties of materials are also governed by their electronic structures, which play a central role in understanding the diverse properties of materials. In the scenario of the ‘flat/steep band model’ for superconductivity [50], the electronic band structures of potential superconductors are expected to possess the feature of steep bands crossing their Fermi level as well as flat bands lying close to the Fermi level. The ‘flat/steep band’ in electronic band structures can be viewed as the functional motif of superconductors, as those found for oxygen-doped Y_2_O_2_Bi [51]. A topological insulator is a material with non-trivial symmetry-protected topological order that behaves as an insulator in its interior but whose surface states are metallic, implying that electrons can only move along the material surface. However, having a conducting surface is not unique to topological insulators, because ordinary band insulators can also support metallic surface states. Topological insulators are unique in that their surface states are symmetrically protected by particle number conservation and time-reversal symmetry [52].

**Figure 8.**
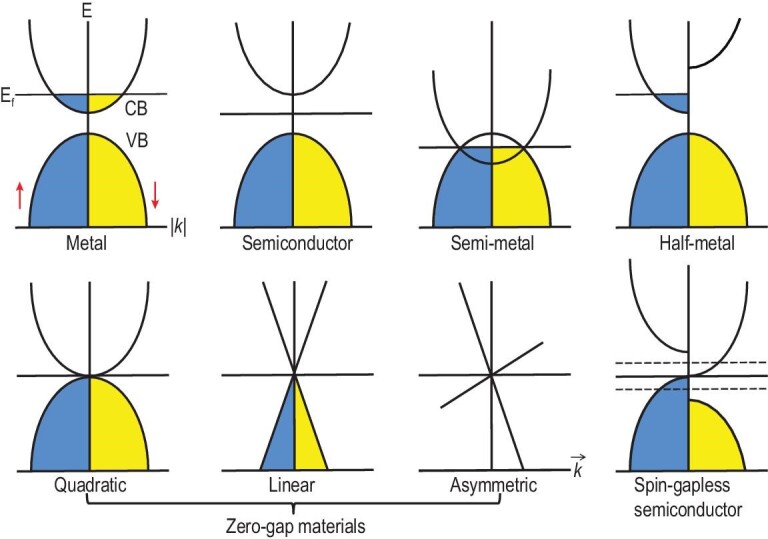
Electronic band structures of various classes of materials in terms of the band dispersions around the Fermi level. Blue and yellow areas indicate the spin-up and spin-down states, respectively. In intrinsic zero-gap materials, the conduction and valence band edges touch the Fermi level. The band structure of spin-gapless semiconductors is dependent on spin. The schemes are based on those in ref. [54].

Figure 8 shows the schematic band structures for various classes of materials. Metals have a partially filled band, indicating that no energy gap exists between the highest occupied and the lowest unoccupied states. By contrast, insulators have a band structure in which the conduction and valence bands are separated by an energy gap (i.e. a band gap). Semiconductors exhibit a small band gap, whereas semimetals have a small overlap between their conduction and valence bands. Half-metals include those that are metallic in one spin direction and semiconducting in the other spin direction. If the conduction and valence band edges meet at the Fermi level, the material belongs to a comparatively new class of solids known as gapless semiconductors or zero-gap materials. Materials with zero-gap band structures can have quadratic, linear and asymmetrical energy vs. *k* dispersions, leading to various states [53].

## FUNCTION-ORIENTED DESIGN OF IR NLO MATERIALS

The various aspects of the microscopic structures of materials, especially those with crucial structure information closely related to the material properties and functions, must be understood in order to design functional materials with desired properties. Therefore, the exploration of functional motifs, which requires uncovering the functional motifs and the role of their arrangements in materials, is important for a function-oriented material design strategy. In principle, no clear distinction exists between structure-oriented or function-oriented strategies, because structure and properties (or functions) are strongly related. If there is any distinction between them, it is that functional motifs in materials are more emphasized in the function-oriented strategy than in the structure-oriented strategy. We consider IR NLO materials [54–56] as examples to elucidate the function-oriented design strategy, with the help of functional motifs.

IR NLO crystals can be used to produce coherent and tunable lights from solid-state lasers, and they have wide application in fields such as medical, military and network communication [57,58]. In general, the prerequisites for IR NLO material candidates include high SHG efficiency, wide optical band gap for high laser-induced damage threshold (LIDT), good IR transparency and the ability to grow bulk single crystals with ease. High NLO efficiency and LIDT are very difficult to achieve together because the NLO efficiency and LIDT are determined mainly by the covalency and ionicity of a compound, respectively, and because the latter two compete with each other [59–61]. The covalent and ionic structural units in IR NLO materials can be viewed as functional motifs for SHG and LIDT performance (i.e. NLO and LIDT functional motifs), respectively. The exploration of IR NLO materials with high NLO efficiency and LIDT should involve a strategy to construct the structural framework of highly oriented NLO functional motifs and combine it with LIDT functional motifs without strong coupling between them in a single compound.

### Exploration of new NLO functional motifs

The anionic group theory (AGT) posits that the overall SHG coefficient of a crystal is the geometrical superposition of the microscopic second-order NLO susceptibility tensors of the relevant anionic group, and it involves no ‘essentially spherical’ metal cations [62]. However, polarizable metal cations strongly contribute to SHG responses [63–65]. Furthermore, the metal cations of NLO compounds can hardly be considered spherical given the non-spherical arrangement of anions surrounding a metal cation. Nevertheless, the AGT provides a useful strategy to search for ultraviolet-visible NLO materials with high NLO responses. Similar to anionic groups, non-spherical cationic groups are expected to have a non-vanishing SHG response. This idea has been confirmed by several examples, such as inorganic supramolecular compounds (Hg_6_P_3_)^3+^(In_2_Cl_9_)^3–^, (Hg_8_As_4_)^4+^(Bi_3_Cl_13_)^4–^, (Hg_6_P_4_Cl_3_)^1+^(PbCl_3_)^1–^ and (Hg_23_P_12_)^12+^[(ZnCl_4_)_6_]^12–^, in which polyanionic and polycationic groups comparably contribute to the overall SHG coefficient and attain large SHG efficiencies comparable to that of AgGaS_2_ [66,67].

**Figure 9.**
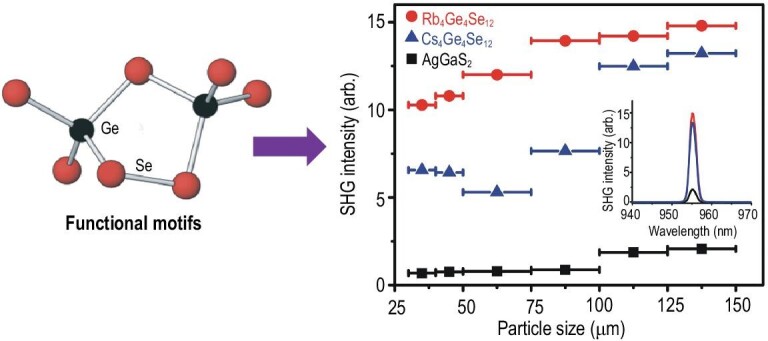
Structure of NLO functional motif [Ge_2_Se_4_(μ-Se_2_)]^4−^ (left). SHG responses of Rb_4_Ge_4_Se_12_, Cs_4_Ge_4_Se_12_ and AgGaS_2_ as a function of particle size (right), where the inset shows the SHG signals of samples with particle sizes of 150–200 μm at the incident laser with a wavelength of 1910 nm and pulse width of 10 ns. Reproduced with permission from ref. [69]. Copyright 2017, American Chemical Society.

Typical IR NLO-active motifs include MQ_4_ (M = Ga, In, Ge, Sn, etc.; Q = S and Se) tetrahedra, which exist in many diamond-like IR NLO materials and contribute dominantly to NLO efficiency. They are typical NLO functional motifs in IR NLO materials. In past decades, many attempts have been made to explore new non-tetrahedral NLO motifs, including the dimeric [Ge_2_Se_4_(μ-Se_2_)]^4−^ unit in A_4_Ge_4_Se_12_ (A = Rb, Cs; Fig. 9) [68], (HgSe_3_)^4–^ in BaHgSe_2_ [69], (HgI_2_Cl_2_)^2−^ in Cs_2_HgI_2_Cl_2_ [70] and (As_3_S_6_)^3−^ in AMnAs_3_S_6_ (A = Cs, Rb) [71]. A general principle for partitioning a structure and its physical property in terms of a set of atoms and bonds was developed, which provides a quantitative ansatz to find the appropriate functional motifs, such as those of NLO crystals [72,73].

### Construction of structural frameworks with highly oriented NLO functional motifs

**Figure 10.**
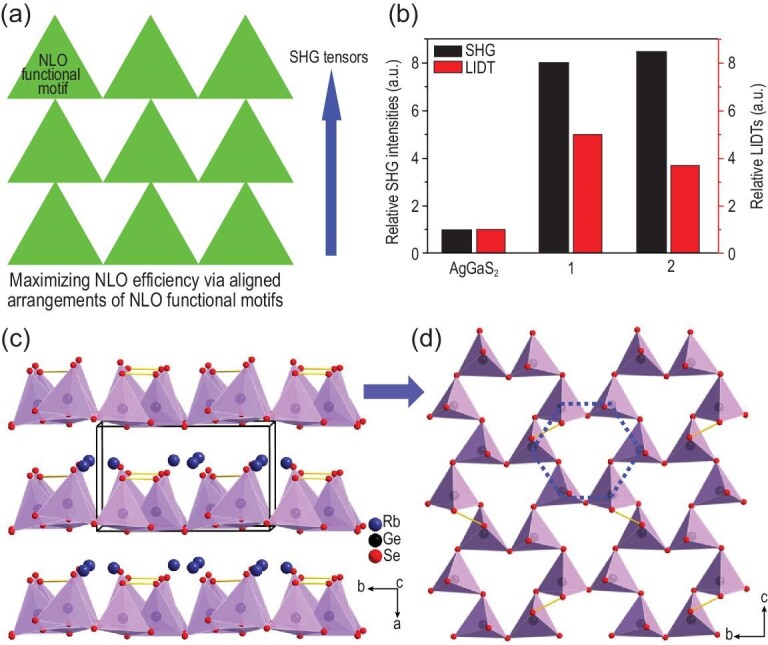
(a) Schematic of the strategy for maximizing macroscopic NLO efficiency by using the aligned arrangements of NLO functional motifs in a sandwich-like structure. (b) Relative LIDTs and SHG intensities of Rb_2_Ge_4_Se_10_ and Cs_2_Ge_4_Se_10_ in comparison with those of AgGaS_2_. (c and d) Structures of Rb_2_Ge_4_Se_10_ viewed along with the *c* and *a* axes in the left and right diagrams, respectively. Cs_2_Ge_4_Se_10_ is isostructural to Rb_2_Ge_4_Se_10_. Reproduced with permission from ref. [75]. Copyright 2018, WILEY-VCH Verlag GmbH & Co. KGaA, Weinheim.

According to the additivity principle of the microscopic second-order susceptibility tensor for NLO-active functional motifs, highly oriented NLO functional motifs have larger net second-order susceptibility tensors compared to a group of NLO functional motifs with random orientations (Fig. 10). Several representative examples have been found. The polyselenides Rb_2_Ge_4_Se_10_ and Cs_2_Ge_4_Se_10_ exhibit a sandwich-like structure, which consists of 2D infinite ^2^_∞_[Ge_4_Se_10_]^2−^ layers made up of highly oriented distorted GeSe_4_ tetrahedra, and large SHG signals (8.0 and 8.5 times that of commercial AgGaS_2_). These results can be ascribed not only to the enhancements of the static contribution from the high orientation of the local dipole moment of the GeSe_4_ tetrahedra but also to the induced enhancement of the contribution from the terminal and Se-bonded Se atoms [74]. In the salt-inclusion sulfide Li[LiCs_2_Cl][Ga_3_S_6_], with a wide band gap of 4.18 eV, the highly orientated GaS_4_ tetrahedra in the [Ga_3_S_6_]^3–^ host are responsible for its good SHG efficiency [75]. Similarly, the SHG efficiency of the NLO materials [ABa_2_Cl][Ga_4_S_8_] (A = Rb, Cs) [76] and [ABa_3_Cl_2_] [Ga_5_S_10_] (A = K, Rb, and Cs) [77] is mainly contributed by the NLO functional motif of GaS_4_ tetrahedra.

### Combination of NLO and LIDT functional motifs

**Figure 11.**
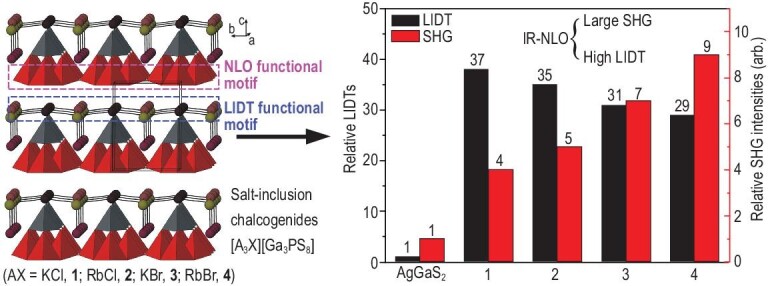
Overview of 3D frameworks of [A_3_X][Ga_3_PS_8_] (A = K, Rb; X = Cl, Br) and their relative LIDTs and SHG intensities in comparison with those of AgGaS_2_. The [Ga_3_PS_10_]^6–^ cluster layers in the structures are the main source of SHG efficiency and they can be viewed as NLO functional motifs. The [A_3_X]^2+^ salt layers mainly contribute wide band gaps and large LIDTs, and they can be viewed as LIDT functional motifs. Reproduced with permission from ref. [79]. Copyright 2016, The Royal Society of Chemistry.

IR NLO materials for practical use must exhibit a high NLO efficiency and a high LIDT. The NLO efficiency of a material can be enhanced by introducing NLO-active covalent structure units with large microscopic SHG susceptibilities. Although the laser damage on NLO crystals is a highly complicated process, LIDT is mainly determined by band gaps and the ionicity of a compound. Therefore, it can be enhanced by introducing ionic structural units such as alkali or alkali-earth metal halides. NLO and LIDT functional motifs can be reasonably combined in a single compound to pursue IR NLO materials with balanced NLO efficiency and LIDT. Salt-inclusion chalcogenides are typically constructed using covalent metal chalcogenide frameworks as hosts and ionic alkali/alkaline-earth metal halide sublattices as guests. These chalcogenides provide great opportunities for exploring IR NLO materials with good comprehensive performance. The salt-inclusion chalcogenides [A_3_X][Ga_3_PS_8_] (A = K, Rb; X = Cl, Br) constructed from an alternate stacking of adamantane-like [Ga_3_PS_10_]^6–^ cluster layers and cationic [A_3_X]^2+^ salt layers present higher SHG responses and higher LIDTs than commercial AgGaS_2_ (Fig. 11) [78].

## DISCUSSION

Understanding the microscopic structures of materials is crucial for uncovering material functional motifs and the arrangements governing material properties, because the latter determine the structure–property relationships of materials. In past decades, numerous breakthroughs and milestones have been achieved in the field of material microscopic structures; these achievements include material structural theories, measurement methods and instruments, and software for structure determination and refinement. These advances help accumulate experimental structure data efficiently. High-throughput experimentation allows the execution of a great number of experiments in parallel. High-throughput experimentation can result in more unified data on material structures and properties with less cost per experiment than the traditional means of experimentation [79].

Computation plays an important role in the field of material research. Two main computation strategies can be used to extract the functional motifs in materials and build structure–property relationships on the basis of the big data of material structures and properties. The first one is high-throughput calculations based on density functional theory (DFT) [80], molecular dynamics [81], Monte Carlo techniques [82], the phase-field method [83] and finite element analysis [84]. DFT calculations can predict material properties with relatively high precision. In most DFT calculations, no additional experimental data are needed except for material structures. For certain types of materials, crystal structures can be predicted from *ab-initio* calculations on the basis of particle swarm optimization algorithms with only chemical compositions as input parameters [85]. Although considerable progress has been made in the field of material calculations in the past few decades, designing materials by theoretical calculations alone remains difficult, mainly due to the limits of fundamental assumptions on which most calculations are based. DFT does not properly consider the many-body correlations, and underestimates band gaps, which affect the properties that require energy differences between occupied and unoccupied states, e.g. the SHG response of NLO materials. Other methods, such as GW approximation, can correct the problem with significantly improved precision [86], but GW calculations are notably time consuming and difficult to apply in high-throughput computations.

DFT calculations for the physical properties of a material involve two steps; the determination of its electronic structure until the self-consistent-field (SCF) iteration is converged, and the calculation of physical properties by using the converged electronic structure. In each of these steps, certain approximations and assumptions are introduced to make the computations possible and manageable. The electron density distribution of a material can, in principle, be determined experimentally. The involvement of electron density, wavefunction and the density matrix in crystallography can be termed as ‘electronic structure crystallography’, which focuses on how to extract the electronic structure information and relevant physicochemical properties of materials on the basis of the theories and methods in X-ray crystallography and quantum chemistry. Different experimental datasets, such as X-ray diffraction and directional Compton scattering, can be used in electronic structure refinements. Polarized neutron diffraction (or magnetic X-ray diffraction) and magnetic Compton scattering can be used for extracting spin-resolved electron density. Usually, high structural resolution and high-quality datasets are required for reliable electronic structure refinements.

The second computation strategy is data mining, which uses artificial neural networks or Bayesian networks to undertake machine learning of material datasets with known structures and properties. The derived numerical network model is then used to predict the properties of known structures and to screen materials with desired properties [87–89]. Data mining is notably faster than DFT calculations, although it needs substantial amounts of reliable data to train the network model. The neural network model is a mathematical model that is used to describe the structure–property relationships of certain types of materials, and it can be refined during machine learning. Given that machine-learning algorithms are usually treated as black boxes and neural network models are complicated to understand analytically, extracting the functional motifs in materials and the structure–property relationships from data mining is a big challenge. Overcoming this challenge is important in future research. In general, the more data used in machine learning, the more reliable the predictions that the model generates. However, for various types of materials, especially those in the early development stages in the fields of material science, adequate experimental data on structures and properties are usually unavailable. DFT calculations are an alternative to experimental studies, and they can be efficiently used to create big data for efficient data mining. Such a strategy was carried out on the electron density of materials with considerable results [90–92]. However, the training models from theoretical data determined by calculations are generally meaningful only for theoretical predictions. Developing complete and reliable databases for structures and properties of a massive number of materials is extremely important [93].

## CONCLUDING REMARKS

In this work, the identification of functional motifs and the examination of their arrangements in materials were discussed. The functional motifs in materials are the critical structural units (e.g. constituent components and building blocks) that play a decisive role in generating material functions, and they can not be replaced with other structural units without resulting in the loss or significant suppression of relevant functions. The arrangements of functional motifs in materials were elucidated by analyzing the microscopic structures of materials covering the main structural aspects of most intrinsic material properties. Functional motifs and their arrangements govern material properties synergistically. This functional motif exploration is crucial for understanding how material properties are related to structures; it enables the rational design of new materials with desired properties. Machine learning is expected to be useful for efficiently predicting material properties and screening materials with desired properties. For the design of new materials, developing sufficiently reliable databases of material structures and properties and new effective methods of extracting functional motifs and structure–property relationships of materials from machine learning models is imperative.
